# Dehalogenating *Desulfoluna* spp. are ubiquitous in host-specific sponge microbiomes of the Great Barrier Reef

**DOI:** 10.1093/ismejo/wraf113

**Published:** 2025-05-31

**Authors:** Lauren A Hall, Katherine D Scott, Nicole Webster, Lee J Kerkhof, Max M Häggblom

**Affiliations:** Department of Biochemistry and Microbiology, Rutgers, the State University of New Jersey, New Brunswick, NJ 09801, United States; Department of Biochemistry and Microbiology, Rutgers, the State University of New Jersey, New Brunswick, NJ 09801, United States; Australian Institute of Marine Science, Townsville, QLD 4810, Australia; Australian Centre for Ecogenomics, University of Queensland, St Lucia, QLD 4072, Australia; Institute for Marine and Antarctic Studies, University of Tasmania, TAS 7001, Australia; Department of Marine and Coastal Sciences, Rutgers, the State University of New Jersey, New Brunswick, NJ 09801, United States; Department of Biochemistry and Microbiology, Rutgers, the State University of New Jersey, New Brunswick, NJ 09801, United States

**Keywords:** sponge microbiome, organohalide respiration, dehalogenation, nanopore long-read sequencing, rRNA operon

## Abstract

Marine sponge holobionts are important contributors to numerous biogeochemical cycles, including the natural organohalogen cycle. Sponges produce diverse brominated secondary metabolites, which select for a population of anaerobic debrominating bacteria within the sponge body. Sponge microbiomes can be host-specific, but the selection and host-specificity of debrominating bacteria are unknown currently. In this study, we used nanopore long-read sequencing of nearly full-length ribosomal RNA operons to evaluate host-specificity of the Great Barrier Reef sponge microbiomes at the strain level and to determine if host specificity extends to sponge-associated dehalogenating bacteria. Reductive debromination activity was observed in anaerobic enrichment cultures established from all Great Barrier Reef sponges. Even though other bacterial symbionts of interest, including *Nitrospira* spp. and Ca. Synechococcus spp. demonstrated strong host-specificity, *Desulfoluna* spp., a key sponge-associated dehalogenating bacterium showed no evidence of host-specificity. This suggests different modes of transmission and/or retention of different members of the sponge microbiome residing within the same host species. These findings expand our understanding of how sponge microbiomes are assembled and the relationship between the host and individual bacterial strains.

## Graphical Abstract

**Graphical Abstract:**
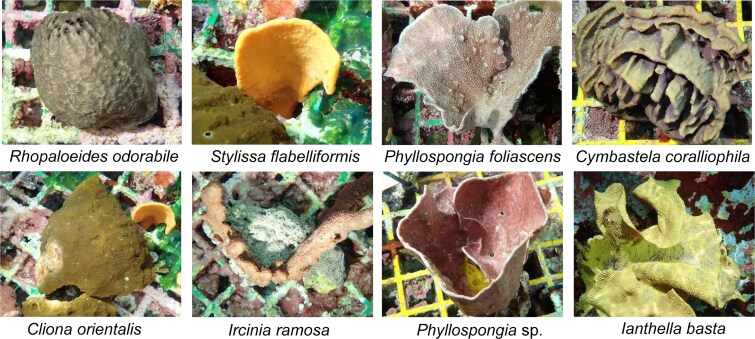

## Introduction

Sponges (*Porifera*) are ancient, filter-feeding animals that are globally distributed in both marine and freshwater environments [1]. Sponges and their microbiota, that together form the sponge holobiont, are abundant and ecologically important residents of coral reefs, contributing to nutrient cycling and providing structural complexity [2–6]. For example, the circulation of seawater through sponge aquiferous systems results in the transformation of dissolved organic matter into forms that other marine species can use in a pathway called the “sponge loop” [7]. Through this loop, organic carbon and other nutrients remain within coral reefs instead of being exported to the open ocean [7].

An overlooked biogeochemical cycle in sponges is that of halogens, and the biotransformation of organohalide compounds. Sponges are a particularly rich source of biogenic organohalides, especially organobromine compounds. A vast array of bioactive organohalide compounds, including phenolics, pyrroles, dioxins, and fatty acids are produced as secondary metabolites by different sponge species and their symbionts [8–10]. These organohalide compounds can resemble anthropogenic pollutants of concern, including brominated flame retardants such as tetrabromobisphenol A and certain polybrominated diphenyl ethers [11]. The brominated metabolites appear to select for a population of dehalogenating bacteria that resides and is active within the sponge animal [12–16]. For example, *Desulfoluna spongiiphila* is the predominant debrominating species found in sponges, forming a cosmopolitan group present in diverse, globally distributed marine sponges [15]. The host-specificity of *Desulfoluna* strains remains unclear, however, and no other organohalide-respiring bacteria have been enriched or isolated from marine sponges to date. Further, sponge-associated dehalogenating bacteria may represent an untapped resource for marine pollution remediation. Given that organohalide contaminants are ubiquitous, recalcitrant, and pose severe health risks, it is of interest to identify and characterize microbes capable of dehalogenation that may be useful for bioremediation [17–20]. Marine sponge holobionts have been proposed as bioremediation tools for several types of contaminants, but their potential use for removal of organobromide contaminants has not been addressed [21].

Beyond dehalogenating bacteria, sponges harbor diverse microbial communities, including obligate symbionts that play important roles in nutrient cycling [4, 22, 23]. Prior studies have primarily employed 16S rRNA gene amplicon sequencing to determine sponge microbiome community structure, typically achieving phylum-level and occasional genus- or species-level resolution [24–29]. These sequencing methods often utilize the V3-V4 hypervariable region of the 16S ribosomal RNA (rRNA) gene. However, this approach does not provide strain-level resolution. Metagenomic analysis of sponge microbiomes has also been performed to explore functional gene diversity and species-level host specificity, but strain-level diversity has not been explored [23, 30–33].

Here, we used rRNA operon sequencing via the Oxford Nanopore Technologies (ONT) MinION to achieve species- and strain-level resolution of the sponge microbiome, offering insights into the assembly and functions of microbial communities [34–40]. The long-read capabilities of the MinION allow for sequencing of near-complete rRNA operon amplicons (~4200 bp), with each read including the near full-length 16S rRNA gene, internal transcribed spacer (ITS) region, and most of 23S rRNA gene. This method can provide strain- and species-level resolution and identification due to the inclusion of the ITS region, resulting in a more detailed analysis of the microbial community [38, 41]. The accuracy of MinION sequencing has improved dramatically, reaching above 96% for R9 chemistry [41], and continues to improve with updated R14 chemistry and basecalling software. Furthermore, raw MinION rRNA operon reads can be used to construct long-read consensus sequences for phylogenetic analysis, allowing for further differentiation of strains that may match to the same entry in the rRNA operon database [34, 35, 37, 42].

To better understand the host-specificity and diversity of sponge-associated microbes and determine whether anaerobic debrominating bacteria are specific to a particular sponge, we compared the microbial communities in a set of eight sponge species from the Great Barrier Reef. Anaerobic organobromine-respiring bacteria were enriched from sponge biomass with 2,6-dibromophenol as electron acceptor. MinION sequencing of rRNA operons was used to analyze sponge-associated prokaryotic communities at the strain level directly from sponge biomass and after enrichment on the brominated substrates. We compared the overall bacterial communities and select bacterial strains of interest among different sponge species living in proximity within the same reef, among individuals within the same species, and from separate segments of the same individual. We expected sponge microbiomes, including debrominating bacteria, to be host-specific and distinct at the strain level. This study provides insights into sponge-microbe interactions and modes of transmission of sponge-associated bacteria.

## Materials and methods

### Sponge collection and biomass dissection

Three individuals each of *Cliona orientalis* (CLI), *Phyllospongia* (formerly *Carteriospongia*) *foliascens* (CAR), *Cymbastela coralliophila* (CYM), *Ianthella basta* (IAN), *Ircinia ramosa* (IRC), *Phyllospongia* sp. (PHY), *Rhopaloeides odorabile* (RHO), and *Stylissa flabelliformis* (STY) were collected from two sites of Backnumbers Reef ~5 km from each other in the Great Barrier Reef in December 2019 (Fig. S1). RHO and STY were sampled from site 1 (18° 31.160 S / 147° 7.557 E) and CLI, CAR, CYM, IAN, IRC and PHY were sampled from site 2 (18° 29.141 S / 147° 9.447 E). Sand samples were also collected from the reef. Sponges were maintained in an aquarium at the Australian Institute for Marine Science (Townsville, Australia) for ~4 weeks before sample preparation. Three replicate biomass samples were dissected from each individual sponge animal and homogenized with a pestle in a 10 ml Falcon tube. Instruments were cleaned with 80% ethanol and rinsed three times with sterile MilliQ water between each sample to avoid cross-contamination. Reef sand samples were processed using the same aseptic techniques. Intact and homogenized sponge biomass and reef sand samples were stored at −80°C until DNA extraction.

### Debrominating enrichment cultures

The anaerobic medium used to enrich for anaerobic debrominating bacteria was adapted from previously published work [43]. Anaerobic minimal salts medium (MSM) amended with 2,6-dibromophenol (2,6-DBP; Sigma-Aldrich Chemical Co., Milwaukee, Wis., USA, min. 99% purity) to a final concentration of 250 μM as the sole terminal electron acceptor was used to enrich for debrominating bacteria. Cultures that did not respond strongly to 2,6-DBP as electron acceptor were transferred into media amended with ethyl 4-bromopyrrole-2-carboxylate (E4B; Sigma-Aldrich Chemical Co., Milwaukee, Wis., min. 97% purity) to the same concentration of 250 μM for further enrichment. Sodium acetate, lactic acid, and sodium propionate added to a concentration of 500 μM (each) served as electron donors and carbon sources. MSM was dispensed in 10 ml aliquots into serum vials, capped with rubber stoppers, sealed with aluminum crimp seals, and sterilized by autoclaving.

One ml of homogenized sponge biomass was inoculated into 10 ml of MSM. Cultures were incubated at room temperature (~22°C) in the dark without shaking. Sterile, uninoculated vials of medium were used as controls to monitor for any abiotic loss of 2,6-DBP. One ml samples were collected every 6-8 weeks before transfer, centrifuged to pellet biomass, and 0.7 ml of supernatant was removed before storing the pellet at −80°C. Supernatant was filtered through a 0.45 μm filter into high-performance liquid chromatography (HPLC) vials.

### Analytical methods

Debromination activity was monitored by HPLC on an Agilent 1100 Series (Agilent Technologies Santa Clara, California, USA) with a C18 column (Hydro-RP phase; 250 × 4.6 mm; particle size, 4 μm; Phenomenex, Torrance, California) using an eluent of 70% methanol, 29% MilliQ water, and 1% acetic acid at a flow rate of 1 ml/min. The UV detector wavelength was set to 254 nm. Standard curves were determined for each analysis set using 2,6-DBP, 2-bromophenol, and phenol standards at the following concentrations: 31.25 μM, 62.5 μM, 125 μM, 250 μM, and 500 μM. Sample concentrations were calculated using the regression line equation of each standard curve.

### DNA extraction and purification

DNA was extracted using a guanidinium thiocyanate method. Briefly, 50 μl of sponge homogenate was combined with 50 μl of Solution 1 (50 mM glucose, 10 mM EDTA, 25 mM Tris-Cl (pH 8.0)) and subjected to four rapid freeze–thaw cycles (−80°C to 55°C). After the final thaw, 35 μl of 4 mg/ml lysozyme in Solution 1 and 15 μl of 0.5 M EDTA were added to the homogenate and incubated at room temperature for 5 minutes. Then, 150 μl of lysis buffer (4 M guanidinium thiocyanate, 10 mM Tris, 50 mM EDTA, 1% SDS) was added followed by a 10 minute incubation at 55°C. DNA was precipitated by adding 100 μl each of 5 M NaCl and 30% PEG/1.5 M NaCl, 2 μl of glycogen, and 1 ml of 100% ethanol. DNA was pelleted by centrifugation and resuspended in 50-100 μl of 10 mM Tris. Extracts with high RNA content were treated with RNase A for 10 minutes prior to AMPure bead (Beckman Coulter, Indianapolis, IN, USA) purification. Following purification, the DNA was stored at −80°C.

### Ribosomal RNA operon amplification

Ribosomal RNA operons were amplified by polymerase chain reaction (PCR) using bacterial domain-specific forward primers ([ONT barcode]- 16S-27F: 5′-AGA GTT TGA TCC TGG CTC AG-3′) and reverse primers ([ONT barcode]- 23S-2241R: 5′-ACC GCC CCA GTH AAA CT-[ONT barcode]-3′) [37]. Each reaction contained 1 μl of purified template DNA (<10 ng), primers, PrimeSTAR GXL *Taq* polymerase (TaKaRa Bio, Ann Arbor, MI, USA), PCR buffer, and dNTPs. Initial denaturation occurred for 3 minutes at 94°C, followed by 25-30 cycles of denaturation for 10 seconds at 98°C, primer annealing for 15 seconds at 60°C, and extension for 5 minutes at 68°C to generate sufficient amplicons for sequencing. After a final extension for 7 minutes at 68°C, PCR product was cooled to 4°C. PCR product was stored at −20°C. rRNA operon amplicons were visualized and quantified by agarose gel electrophoresis.

### Library preparation and sequencing

Libraries were prepared using the SQK-LSK109 sequencing kit (Oxford Nanopore Technologies Ltd., Oxford, United Kingdom). Fifty ng each of up to 36 barcoded rRNA operon amplicons were combined into a library. Libraries were cleaned using an AMPure bead (Beckman Coulter, Indianapolis, IN, USA) purification protocol. End repair and dA-tailing were done according to ONT instructions using New England Biolabs (Ipswich, MA, USA) kits followed by bead cleaning as above. Ligation of the ONT adaptor was done using the NEB Blunt/TA master mix and 2 μl of fresh 4 mg/ml ATP. The library was again bead cleaned per the kit instructions. Libraries were sequenced using R9 flow cells. Guppy 3.2.2 was used for basecalling, and QA/QC was performed using Geneious R11. Briefly, sequences with lengths between 3700 bp and 5700 bp were retained and sorted by barcode prior to informatic analysis. Raw sequence data and associated metadata are deposited in GenBank with the Bioproject accession no. PRJNA1237858.

### Community analysis

Operon sequences were screened against a ribosomal operon database (rOPDB) [38, https://www.njmicrobe.org/ropdb] using megaBLAST with the following parameters: word size of 60, match/mismatch scoring of 2/−3, gap open cost of 0, and gap extend cost of 4. Sequences with alignments of ≥1000 bp were retained. For all sequences, the ITS region and 23S rRNA genes were trimmed and an additional BLAST search was conducted to screen 16S rRNA genes against SILVA v138.1 [44]. Top hit tables were exported as .csv files. BLAST data were concatenated by sponge individual and relative abundance of families and strains calculated in R (4.2.1) using packages readxl, data.table, dplyr, tidyr, and forcats [45–48]. Relative abundance data was plotted as stacked bar charts and bubble plots using reshape2, ggplot2, and ggnested [49, 50, https://github.com/gmteunisse/ggnested].

### Non-metric multidimensional scaling analysis

BLAST output .csv files were imported into R (4.2.1) and concatenated into a single data frame using the packages readxl, data.table, dplyr, and tidyr [45–48]. Relative abundance of strains was calculated for each sample to normalize for variation in total read counts. The package vegan [51] was used to construct a dissimilarity matrix using Bray–Curtis dissimilarity scores. The final NMDS plot was generated from the dissimilarity matrix using R packages vegan and ggplot2 [50, 51]. The stress value for 2-dimensional analysis was calculated using the metamds() function and the stress plot constructed using the stressplot() function, both in vegan [51].

**Figure 1:**
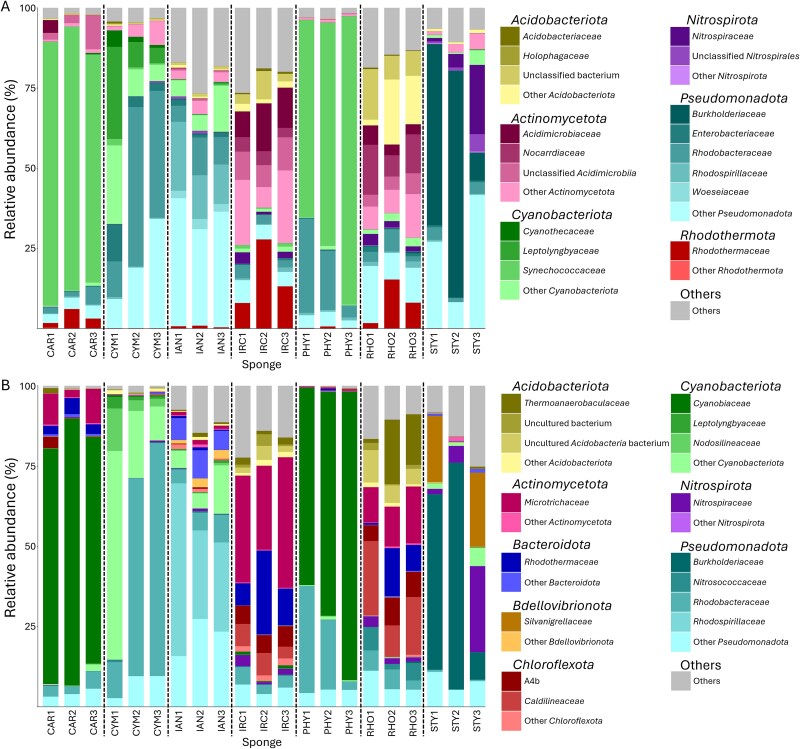
Most abundant bacterial phyla codetected in sponge tissue extracts of *Phyllospongia foliascens* (CAR), *Cymbastela coralliophila* (CYM), *Ianthella basta* (IAN), *Ircina ramose* (IRC), *Phyllospongia* sp. (PHY), *Rhopaloeides odorabile* (RHO), and *Stylissa flabelliformis* (STY) based on (A) rRNA operon data screened against an rRNA operon database [38] or (B) 16S rRNA gene data screened against Silva v138.1 [44]. (DNA was not recovered from *Cliona orientalis* samples).

### Long-read consensus reconstruction and phylogenetic analysis

Long-read consensus sequences (LRCs) for Best BLAST Hits (BBHs) matching to strains of interest were constructed using LastZ v1.02 [52] in an iterative approach with 50X read coverage. Maximum-likelihood trees were reconstructed using FastTree 2.1 for unambiguously aligned bases of the 16S and 23S rRNA genes from LRCs and closely related strains [53]. *Desulfoluna* have two rRNA operons in their genome, one with tRNA genes in the ITS region and one without [54, 55]. For analysis of the *Desulfoluna* spp. detected in dehalogenating enrichment cultures, LRCs were constructed from operons with no tRNA genes in the ITS region. LRCs constructed from BBHs matching Ca. Synechococcus spongiarum SH4 and operons from closely related strains were used to construct a database, against which the raw reads from *Phyllospongia foliascens* and *Phyllospongia* sp. were re-screened. Relative abundances were calculated, and bubble plot were generated using ggplot2 [50].

## Results

### Strain-level host specificity and diversity of sponge microbiomes

To determine host-specificity of sponge microbiomes and the relative abundance of different bacterial taxa, eight different sponge species (*Phyllospongia* (formerly *Carteriospongia*) *foliascens*, *Cymbastela coralliophila*, *Ianthella basta*, *I. ramosa*, *Phyllospongia* sp., *Rhopaloeides odorabile*, *Stylissa flabelliformis*, and *Cliona orientalis*) were collected from Backnumbers Reef in the GBR, Australia. Triplicate subsamples (segments) from three individuals of each sponge species were separately homogenized, DNA was purified, and the bacterial communities were profiled by rRNA operon sequencing. (*Cliona orientalis* samples were not profiled because DNA was not recovered from the sponge homogenate)*.* The total number of prokaryotic taxa detected in the sponge animals varied between homogenates. Individual sponges harbored between 1358 (*Phyllospongia* sp.) and 3410 (*Stylissa flabelliformis*) unique taxa, representing 923 to 1958 species, respectively. Over 7000 unique prokaryotic strains representing more than 4000 species were detected in one individual of *Ianthella basta*. Members of the *Pseudomonadota* (formerly *Proteobacteria*) the *Cyanobacteriota* (formerly *Cyanobacteria*), the *Actinomycetota* (formerly *Actinobacteria*), and the *Acidobacteriota* (formerly *Acidobacteria*) were the most abundant taxa found in all sponges surveyed. At the family level, sponge-associated bacterial communities were more similar in individuals from the same sponge species than between sponge species (Fig. 1). Analysis of the amplicon reads with the rRNA operon database (rOPDB) and 16S rRNA gene only database (Silva v 138.1) indicated the same patterns in relative abundance of the different prokaryotic families (Figs. 1A and 1B). However, there were some differences in the taxon IDs, depending on whether the database used for screening had updated nomenclature [56]. When screened against the rOPDB, rRNA operon sequence data returned strain-level taxonomy information for most reads. Family-level identification was the lowest taxonomic level that could be reliably obtained from analysis of 16S rRNA gene sequences only.

**Figure 2:**
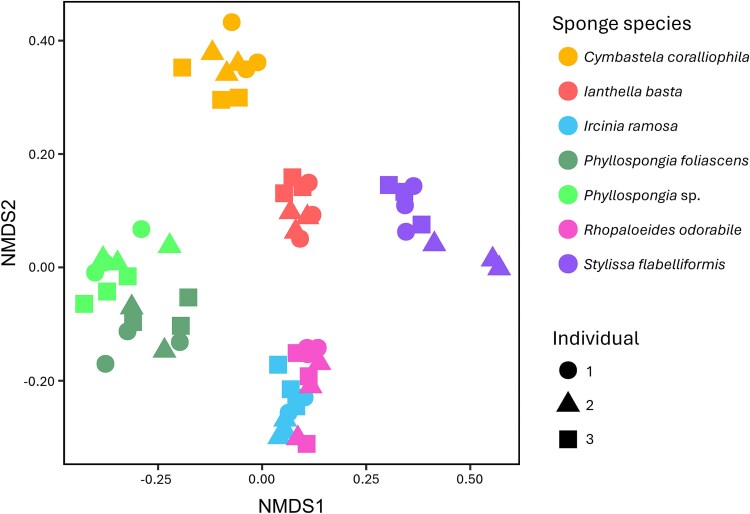
Non-metric multidimensional scaling (NMDS) analysis of sponge-associated prokaryote communities based on Bray–Curtis dissimilarity scores of rRNA operon sequences. DNA extracted from three separate segments of three individual animals of each sponge species was analyzed. The stress value for 2-dimensional analysis was 0.156 (Fig. S2).

**Figure 3:**
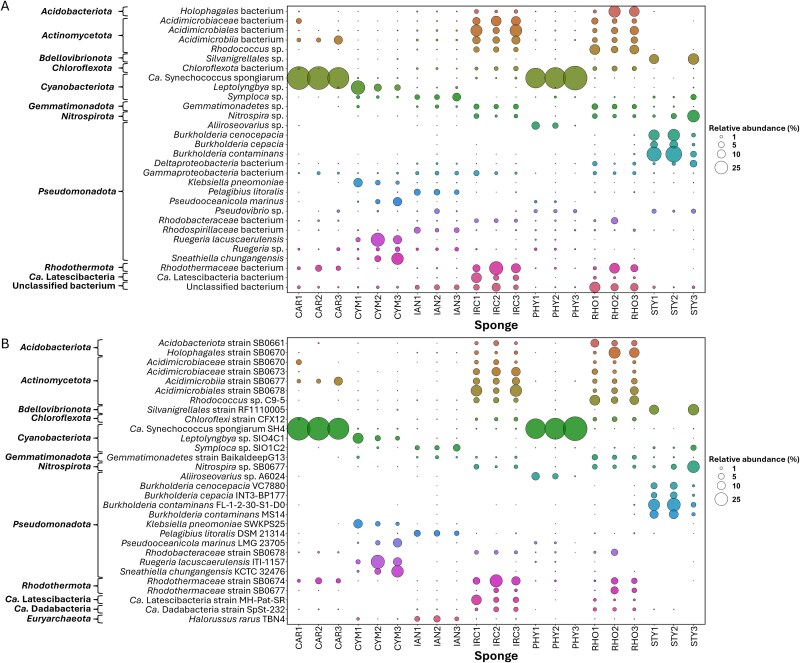
Bubble plots depicting the relative abundances of the 30 most abundant prokaryotic (A) species or (B) strains detected in different sponge species. The data for each sponge individual is the mean of triplicate segment replicates, as minimal variation between segments was seen in NMDS analysis.

Non-metric multidimensional scaling analysis of rRNA operon sequence data (strain-level resolution) demonstrated that the bacterial community composition in subsamples from the same sponge were more similar to each other than to other sponges (Fig. 2; Fig. S2). However, there was overlap between the community composition in the pairs *I. ramosa* – *Rhopaloeides odorabile* and *Phyllospongia foliascens* – *Phyllospongia* sp. The 30 most abundant bacterial species/strains detected were generally consistent across individuals of a sponge species (Fig. 3). Some variation in the relative abundance of strains was detected among different segments of the same individual, suggesting that bacteria are not homogenously distributed in the sponge animal (Figs. S3-S9).

### Dehalogenation activity in sponge enrichments and diversity of *Desulfoluna* spp.

Reductive debromination of 2,6-dibromophenol (2,6-DBP) via 2-bromophenol to phenol was detected in initial anaerobic enrichment cultures established from all sponges (3 individuals of each of the eight species; Table 1). No debromination or loss of 2,6-DBP was detected in the sterile controls. Debromination activity on 2,6-DBP was maintained across multiple sequential 1/10 transfers into fresh medium for all sponge cultures, except for those from *Stylissa flabelliformis*. 2,6-DBP was selected as the terminal electron acceptor for enrichment due to its structural similarities to characterized brominated metabolites produced by several sponges; however, *Stylissa* naturally produces brominated pyrrole-imidazole alkaloids rather than phenolics [57, 58]. A second set of enrichment cultures were established from the *Ianthella* and *Stylissa* sponge samples by transferring 1 ml of the first-generation enrichment cultures on 2,6-DBP into fresh media amended with ethyl 4-bromopyrrole-2-carboxylate (E4B). Dehalogenating activity was detected in both E4B culture sets, but *Stylissa* cultures showed faster loss of E4B and accumulation of the debrominated product than *Ianthella* cultures (data not shown).

**Table 1:** Dehalogenating activity and detection of putative dehalogenating bacteria in sponge enrichment cultures. Initial cultures were amended with 2,6-DBP and maintained through sequential 1/10 transfers of the cultures. Select sponge cultures were amended with E4B to determine dehalogenating activity.

Sponge	Debromination			*Desulfoluna* spp.	*Halodesulfovibrio* spp.
	2,6-DBP		E4B		
	Initial	Sustained	Initial		
*Cliona orientalis*	+	+	n.d.	+	+
*Cymbastela coralliophila*	+	+	n.d.	+	−
*Ianthella basta*	+	+	some	+	+
*Ircina ramosa*	+	+	n.d.	+	+
*Phyllospongia foliascens*	+	+	n.d.	+	−
*Phyllospongia* sp.	+	+	n.d.	+	−
*Rhopaloiedes odorabile*	+	+	n.d.	+	−
*Stylissa flabelliformis*	+	−	+	n.d.	n.d.

+ indicates debromination activity observed in each of the replicate cultures, − indicates no activity, n.d. not determined. Positive detection of *Desulfoluna* and *Halodesulfovibrio* strains was defined as a minimum of 50 rRNA operon reads matching to these genera out of a total of ~3000-15 000 reads in the enrichment culture community.

**Figure 4:**
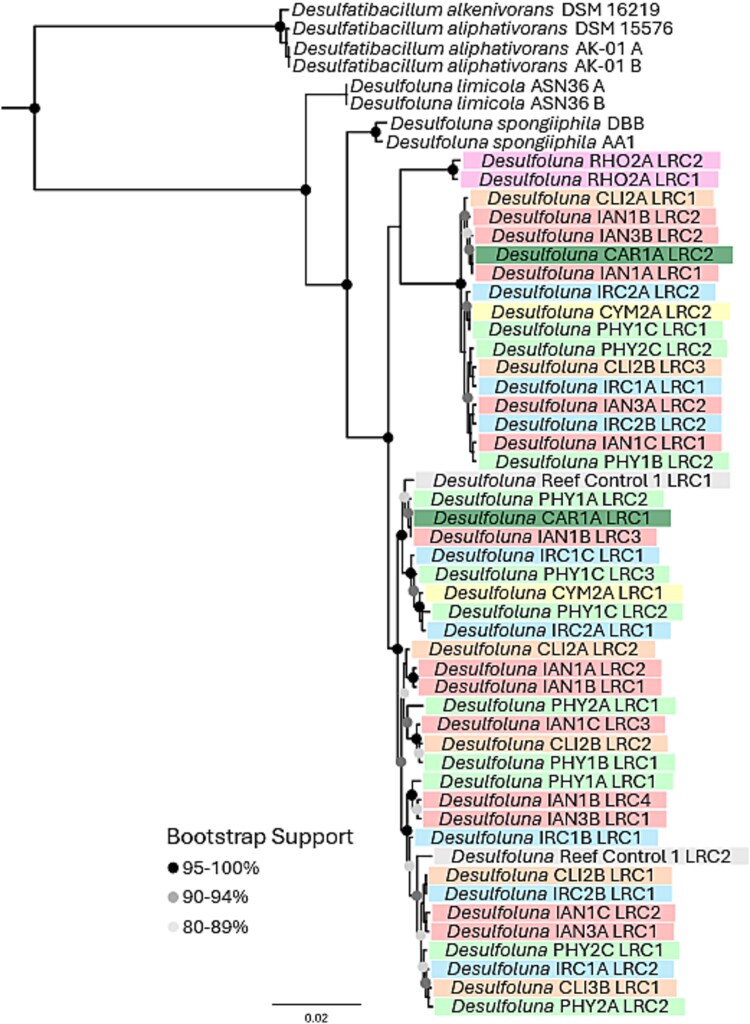
Phylogeny of *Desulfoluna* spp. enriched from GBR sponges and related strains. Maximum-likelihood tree was reconstructed using FastTree 2.1 for 3812 unambiguously aligned base pairs in the 16S and 23S rRNA genes. LRCs are coded by color for the different sponge species from which the strain was enriched. *Desulfatiglans* sp. IK1 and *Desulfococcus oleovorans* Hxd3 were included as outgroups (not shown).

Active debrominating cultures were maintained by sequential 1/10 transfers into fresh medium with lactate as the carbon source and 2,6-DBP as the terminal electron acceptor. The bacterial communities were profiled by rRNA operon sequencing with the aim of identifying (putative) dehalogenating bacteria that were enriched. *Desulfoluna* spp. were detected in all debrominating enrichment cultures, and for most sponge cultures their relative abundance increased with each subculture. In enrichment cultures of some sponges, *Halodesulfovibrio* spp., another putative dehalogenating bacterium, was also detected. Phylogenetic analysis of *Desulfoluna* spp. was performed to determine if there is a strain-specific selection by the different host sponge species. Tree re-construction of *Desulfoluna* LRCs from highly enriched debrominating cultures identified a diverse set of strains that clustered in two main clades (Fig. 4) that likely represent two novel *Desulfoluna* species. However, the strain distribution did not reveal any clear patterns based on host sponge species. All the *Desulfoluna* strains detected in Pacific Ocean sponges and adjacent reef sand diverged from the two previously described Mediterranean *D. spongiiphila* strains AA1 and DBB [13, 55] and *Desulfoluna limicola* MSL71 from sediment of a brackish lake [59]. The GBR strains formed two large clusters with no clear grouping by host sponge species or the reef sand control. Although *Desulfoluna* spp. were enriched from every sponge analyzed, this particular taxon was detected in very low abundance in sponge homogenates, often comprising only a fraction of a percent of the overall community in a sponge segment based on rRNA operon reads (~1-10 *Desulfoluna* spp. reads/100000 rRNA operon reads).

### Phylogeny of *Nitrospira* species and strains

**Figure 5:**
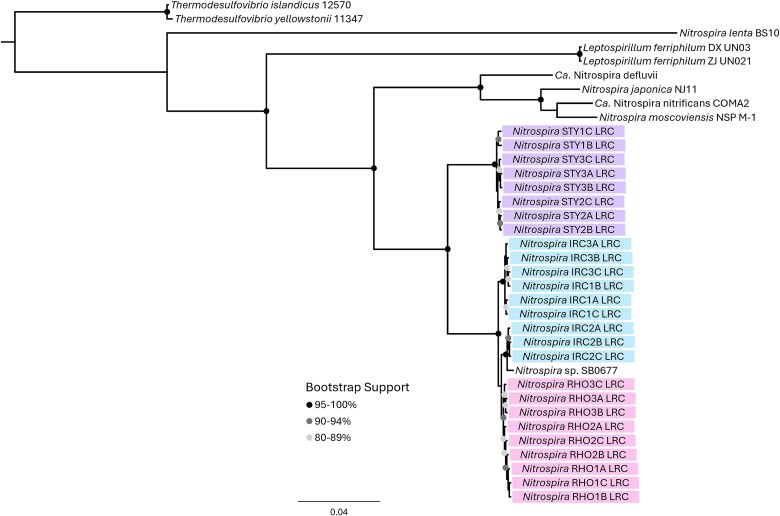
Host-specific *Nitrospira* sp. strains detected in *Ircinia ramosa* (IRC), *Rhopaloeides odorabile* (RHO), and *Stylissa flabelliformis* (STY). LRCs were constructed with 50X coverage from *Nitrospira* sp. SB0677 rRNA operon reads. Maximum-likelihood tree was reconstructed using FastTree 2.1 for 4067 unambiguously aligned base pairs of the 16S and 23S rRNA genes.

*Nitrospira* was detected in *I. ramosa* (IRC), *Rhopaloeides odorabile* (RHO), and *Stylissa flabelliformis* (STY) sponges at higher abundance compared to the other sponges (Fig. 3). rRNA operon reads matched most closely to *Nitrospira* sp. SB0677 and were selected for further analysis due to their ubiquitous nature and the critical contribution of *Nitrospira* to nitrogen cycling in marine systems [60]. LRCs were constructed from rRNA operon reads from three segments of three individuals per sponge species to assess for variability within and between the sponge animals. Phylogenetic analysis of these *Nitrospira* spp. suggests strong host-specificity, as strains detected in the same sponge host were more closely related to each other than they were to strains detected in other hosts, regardless of segment or individual (Fig. 5).

### Diversity of *Synechococcus* strains in *Phyllospongia* sponges

**Figure 6:**
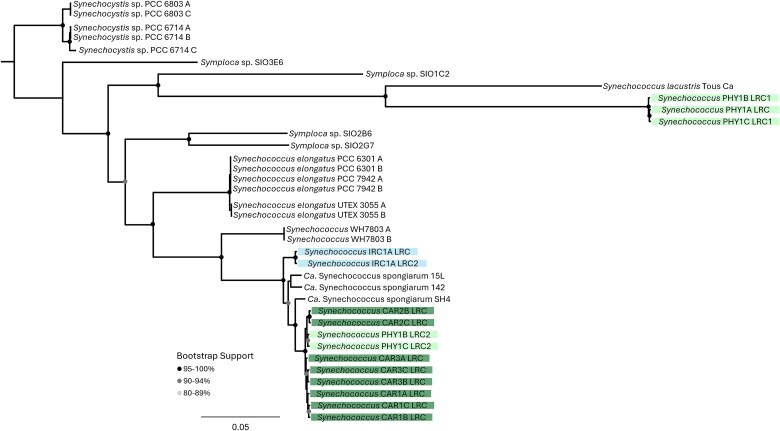
*Synechococcus* strains detected in *Phyllospongia* foliascens (CAR), *Phyllospongia* sp. (PHY), and *Ircinia ramosa* (IRC). LRCs were constructed with 50X coverage from *Candidatus* Synechococcus spongiarum SH4 rRNA operon reads. Maximum-likelihood tree was reconstructed using FastTree 2.1 for 3957 unambiguously aligned base pairs in the 16S and 23S rRNA genes.

**Figure 7:**
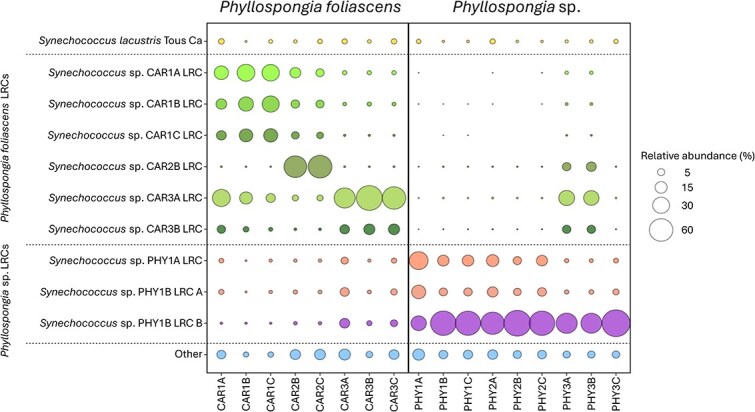
Abundance of different Ca. Synechococcus spongiarum strains detected in segments of sponges *Phyllospongia foliascens* (CAR) and *Phyllospongia* sp. (PHY). Strains appear to be host-specific, with reads matching primarily to LRCs constructed using sequences derived from the same host species.

Previous work has shown that *Phyllospongia* and *Cliona* species bleach in low-light conditions with mixed ability to recover when returned to normal light conditions, establishing an obligate relationship of these phototropic sponges and the *Cyanobacteriota* [61]. The sponges *P. foliascens* (CAR) and *Phyllospongia* sp. (PHY) both harbored abundant *Cyanobacteriota* communities, accounting for over 50% of the total bacterial rRNA operon reads in each individual (Fig. 1). *Cyanobacteriota* were also detected in lower abundance in *I. ramosa* (IRC). The majority of *Cyanobacteriota* rRNA operon reads in both CAR and PHY matched most closely to strain *Candidatus* Synechococcus spongiarum SH4; thus, we constructed LRCs to determine host-specificity of these symbionts. Analysis of LRCs revealed that divergent *Synechococcus* strains were present in CAR, PHY, and IRC (Fig. 6). The analysis of these LRCs showed that the *Synechococcus* strains were mostly sponge-host specific, with CAR reads mainly matching to CAR-derived LRCs, and PHY reads to PHY-derived LRCs (Fig. 7). Ca. Synechococcus spongiarum shows strain-level host specificity, similar to *Nitrospira* spp., in contrast to the apparently stochastic distribution of *Desulfoluna* spp.

## Discussion

Sponge holobionts are major contributors to nutrient cycling in coral reefs and the global ocean [62]. Rates of nutrient cycling in sponges can fluctuate seasonally and are impacted by climate change [63, 64]. Perturbations of the sponge-associated microbiome have been linked to thermal stress and ocean acidification, which may have negative implications for nutrient cycling in ecosystems in which sponges reside [65, 66]. Thus, it is important to understand their microbiome architectures and the factors that influence the microbial community assembly and associated ecosystem functions. By utilizing a near full-length rRNA operon sequencing approach, we gained strain-level information about the diversity, identity, and functions of members of different sponge microbiomes. Here, we emphasize the importance of dehalogenating bacteria and the natural organohalide cycle, in particular the genus *Desulfoluna* which includes the sponge-associated strain *D. spongiiphila* AA1, a known organobromide respirer [54, 67]. These findings may have implications for bioremediation of marine ecosystems contaminated with brominated persistent organic pollutants [14, 15].

Our bacterial community analysis corroborates several other studies [24–26, 29, 68–70] showing host-specificity of the overall microbiome of Great Barrier Reef sponges at all taxonomic levels and with higher degrees of similarity in more phylogenetically similar sponges (Figs. 1 and 3). Indeed, the sponge *Phyllospongia foliascens* was renamed from *Carteriospongia* based on recent morphological and molecular assessment [71], which may explain the high degree of similarity of the *P. foliascens* and *Phyllospongia* sp. resident microbiota. However, *Ircinia* and *Rhopaloeides* are less closely related, belonging to different families within the order *Dictyoceratida* (*Irciniidae* and *Spongiidae*, respectively), but interestingly still harbor similar microbiomes. Minimal variation in bacterial community structure was observed between segments of the same sponge individual and members of the same species confirming the strong host-specificity and assembly (Fig. 2).

Debrominating bacteria were active and could be enriched from every sponge animal tested, similar to what has been observed in our previous studies [12, 15]. Organohalide respiring cultures could be enriched and maintained from the sponge samples as well as a reef sand control. Reductive dehalogenase genes and the capacity for organohalide respiration appears to be widely distributed in different members of marine *Pseudomonadota* (*Deltaproteobacteria*) [72] and even though the medium used does not exclude growth of other 2,6-DBP respiring bacteria, in all of the sponge enrichments, a *Desulfoluna* strain was detected as one of the dominant enriched bacteria. Combined with our previous analysis of Meditterranean and Atlantic sponges [13, 15] the detection of *Desulfoluna* in all of the GBR sponges tested confirms that *Desulfoluna* spp. are cosmopolitan sponge-associated organohalide-respiring bacteria. Two new *Desulfoluna* clades in the GBR sponges were discerned by rRNA operon phylogeny and likely represent novel species. In contrast to the overall sponge bacterial communities that were highly host specific at the strain level, phylogenetic analysis of 2,6-DBP debrominating strains enriched from genetically distant hosts revealed no clear strain-level host-specificity of *Desulfoluna* strains in Great Barrier Reef sponges (Fig. 4). These data suggest that dehalogenating bacteria in these sponges are horizontally transferred or acquired from surrounding seawater rather than vertically transmitted.

Phylogenetic separation of Pacific strains from those isolated from Mediterranean sponges (*D. spongiiphila* AA1 and DBB) [13, 55] or brackish lake sediment (*D. limicola*) [59] lends support to the hypothesis that dehalogenating strains are specific to the location. However, the current study is limited by geographical restriction to the Great Barrier Reef. It is not yet clear whether the stoichastic distribution of *Desulfoluna* spp. is limited to GBR sponges living in relatively close proximity. Previously, *Desulfoluna* spp. (most closely related to *D. spongiiphila*) were detected in Mediterranean sponges and a different *Desulfoluna* sp. was found in a sponge off the coast of Peru [12, 15, 55]. The phylogenetic separation of the two Mediterranean *D. spongiiphila* strains, AA1 and DBB, from the GBR *Desulfoluna* spp. (Fig. 4) suggests geographic separation, but additional sampling and investigation is required to address this question. Though the use of rRNA operon amplicon analysis provides deeper insights into community structure and assembly, it does not provide direct functional information. Additional metagenomic sequencing and analysis of new debrominating isolates will be required to confirm the dehalogenating ability and reductive dehalogenase gene distribution of the novel *Desulfoluna* strains detected here. It will also be of interest to quantify the absolute abundance of *Desulfoluna* spp. in the biomass of different sponges, which may address the potential influence of cultivation bias.

The apparent random distribution of different *Desulfoluna* strains across the GBR sponge species is in contrast with the strong host selectivity of *Nitrospira* and *Synechococcus* spp. Phylogenetic analysis provided evidence for distinct strains in the different sponge hosts (Figs. 6 and 7), implying vertical transmission of these bacteria or some strain selective retention mechanism. In the case of nitrifying bacteria, there is already strong evidence of vertical transmission [73]. Sponges are known to acquire microbial symbionts via both horizontal and vertical transmission [73–79], but the degree to which each mode contributes to the mature sponge microbiome is still debated [68, 80]. Our results support the claim that some bacterial symbionts are selectively retained/vertically transmitted whereas others are horizontally acquired, which may reflect their level of contribution to the overall health of the holobiont. In conclusion, we show that host-specificity of sponge microbiomes can be demonstrated from the phylum to the strain level; however, host-specificity of strains does not appear to extend to dehalogenating *Desulfoluna* symbionts. Although specificity is not seen at the strain level, these data offer additional support to the hypothesis that some members of the genus *Desulfoluna* are sponge-specific dehalogenating species with a cosmopolitan distribution.

## Supplementary Material

Dehalogenating_Desulfoluna_spp_Supplementary_Figures_wraf113

## Data Availability

The rRNA operon reads from Great Barrier Reef sponge samples and enrichment cultures are available in BioProject ID PRJNA1237858. Genbank accession numbers for *Nitrospira* and *Synechococcus* rRNA operon long-read consensus sequences are PV363073-PV363113. Genbank accession numbers for *Desulfoluna* rRNA operon long-read consensus sequences are PV356226-PV356271.
