# Supplementary material for: Dehalogenating Desulfoluna spp. are ubiquitous in host-specific sponge microbiomes of the Great Barrier Reef
Source: ISME J. 2025 May 31;19(1):wraf113. doi: 10.1093/ismejo/wraf113 (PMC12203066; doi:10.1093/ismejo/wraf113)
Supplement: Dehalogenating_Desulfoluna_spp_Supplementary_Figures_wraf113 [file dehalogenating_desulfoluna_spp_supplementary_figures_wraf113.pdf]

SUPPLEMENTARY FIGURES

Dehalogenating *Desulfoluna* spp. are Ubiquitous in Host-Specific Sponge Microbiomes of the Great Barrier Reef

Lauren A. Hall [a], Katherine D. Scott [a], Nicole Webster [b,c,d], Lee J. Kerkhof [e], Max M. Häggblom [a*]

[a] Department of Biochemistry and Microbiology, Rutgers, the State University of New Jersey, New Brunswick NJ 09801, USA

[b] Australian Institute of Marine Science, Townsville QLD 4810, Australia

[c] Australian Centre for Ecogenomics, University of Queensland, St Lucia, QLD 4072, Australia

[d] Institute for Marine and Antarctic Studies, University of Tasmania, TAS, 7001, Australia

[e] Department of Marine and Coastal Sciences, Rutgers, the State University of New Jersey, New Brunswick NJ 09801, USA

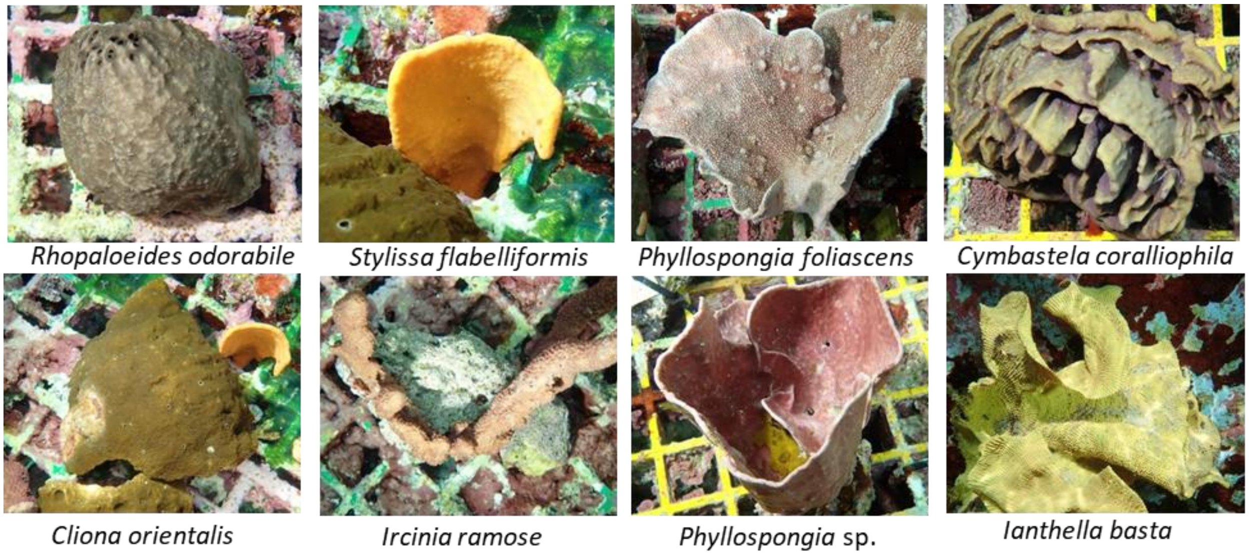

Figure S1: Sponges collected from Backnumbers Reef, Great Barrier Reef.

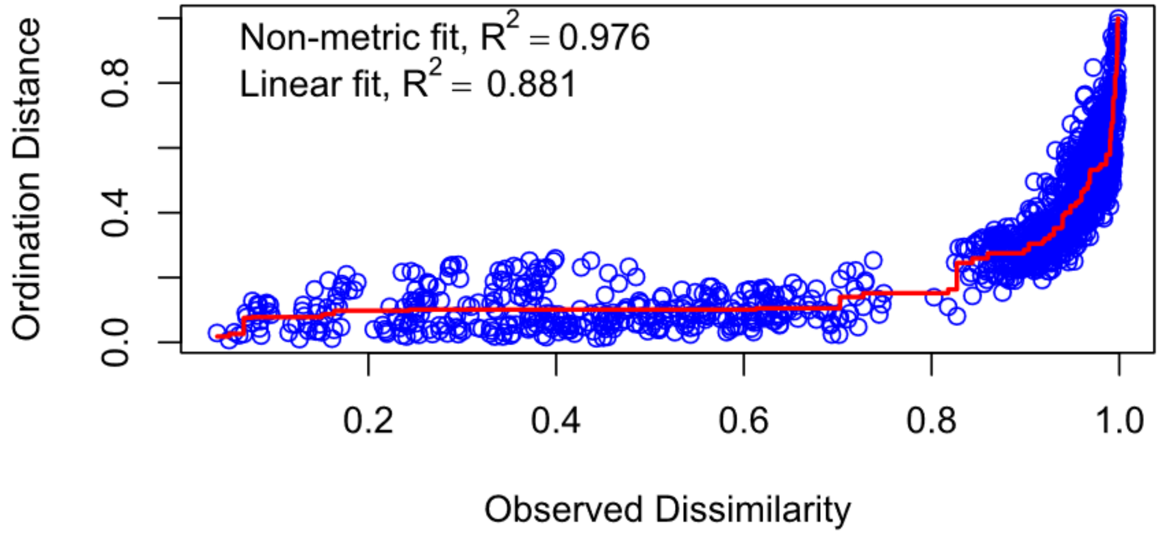

Figure S2: NMDS stress plot to determine goodness of fit.

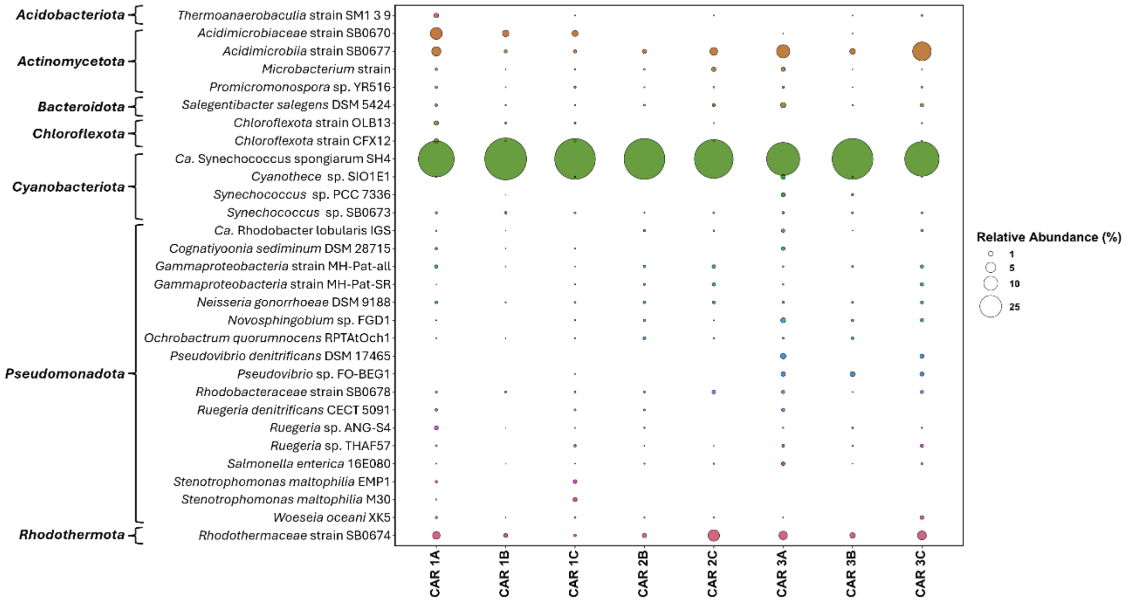

Figure S3: Bubble plot depicting the relative abundances of the 30 most abundant prokaryotic strains in three individuals and segments of *Phyllospongia foliascens*.

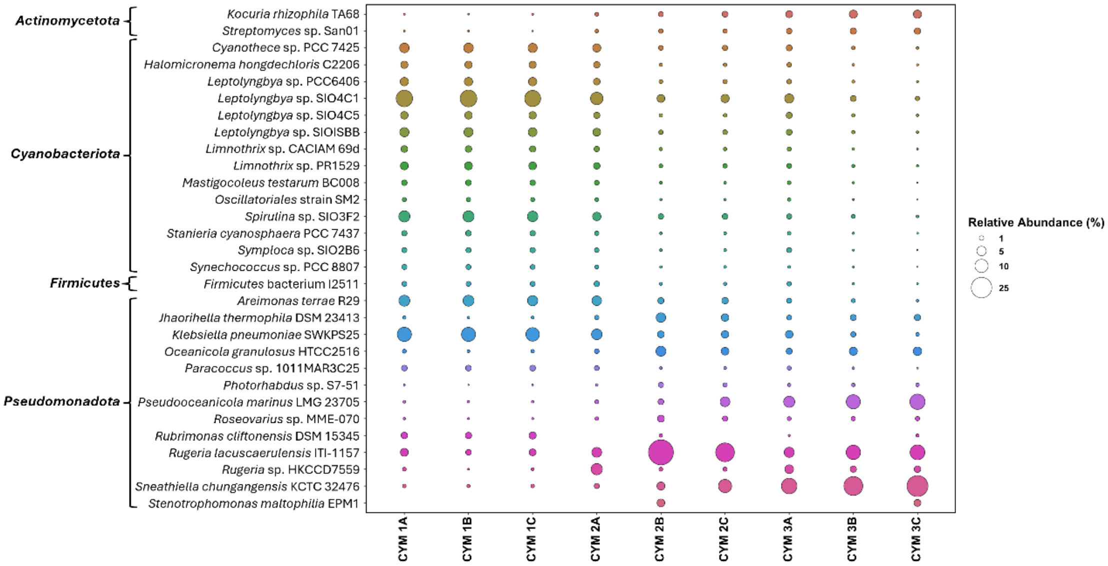

Figure S4: Bubble plot depicting the relative abundances of the 30 most abundant prokaryotic strains in three individuals and segments of *Cymbastela coralliophila*.

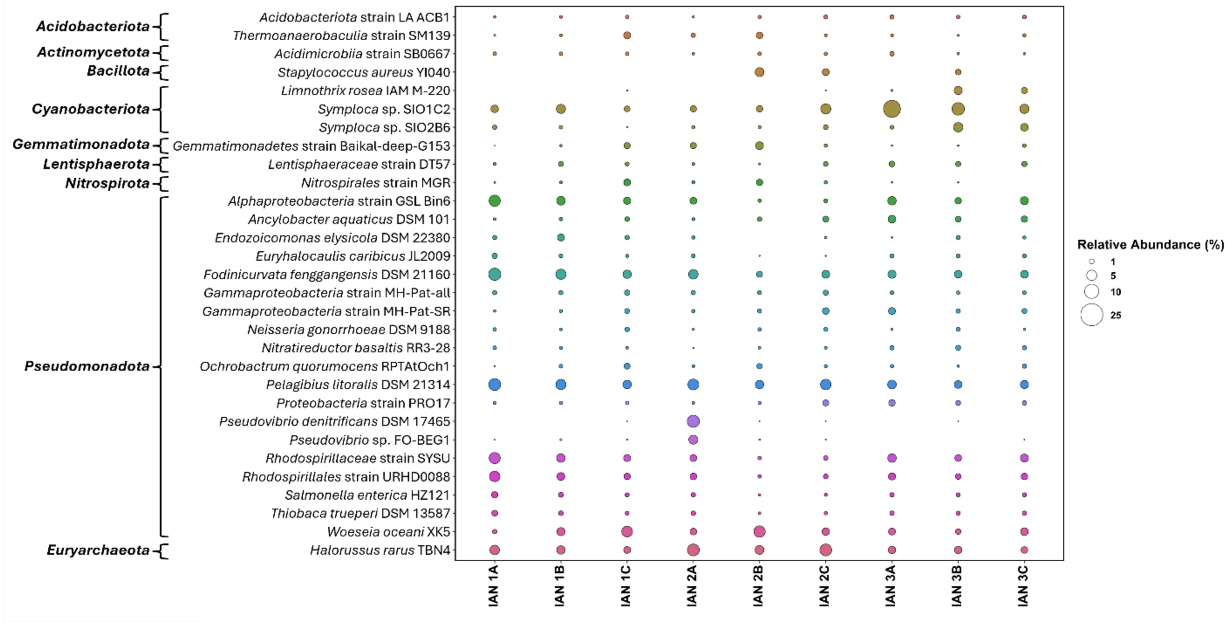

Figure S5: Bubble plot depicting the relative abundances of the 30 most abundant prokaryotic strains in three individuals and segments of *Ianthella basta*.

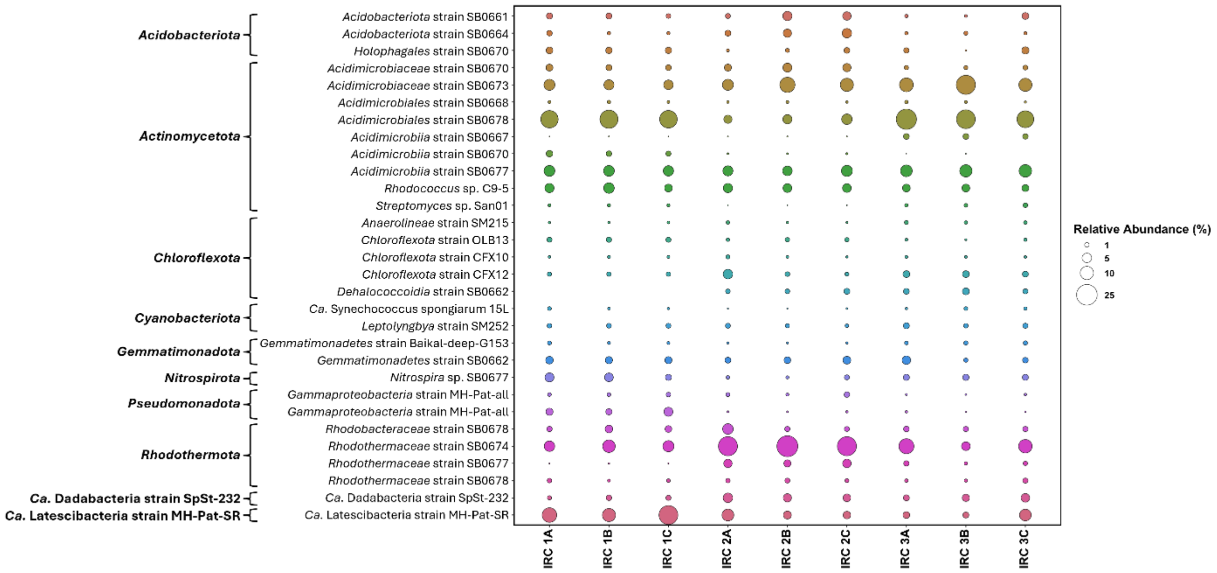

Figure S6: Bubble plot depicting the relative abundances of the 30 most abundant prokaryotic strains in three individuals and segments of *Ircinia ramosa*.

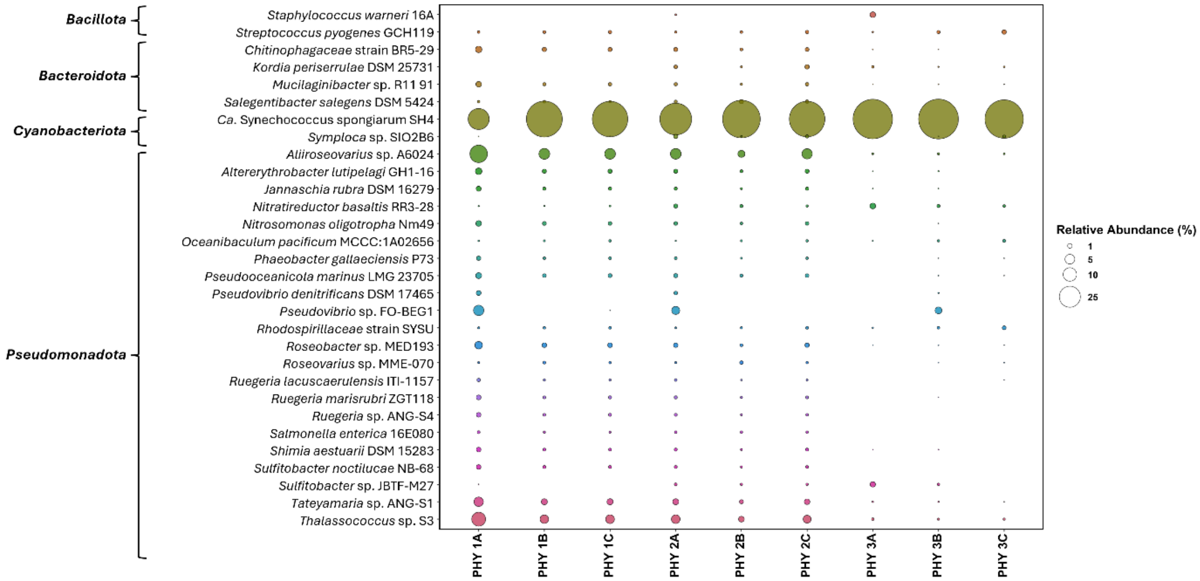

Figure S7: Bubble plot depicting the relative abundances of the 30 most abundant prokaryotic strains in three individuals and segments of *Phyllospongia* sp.

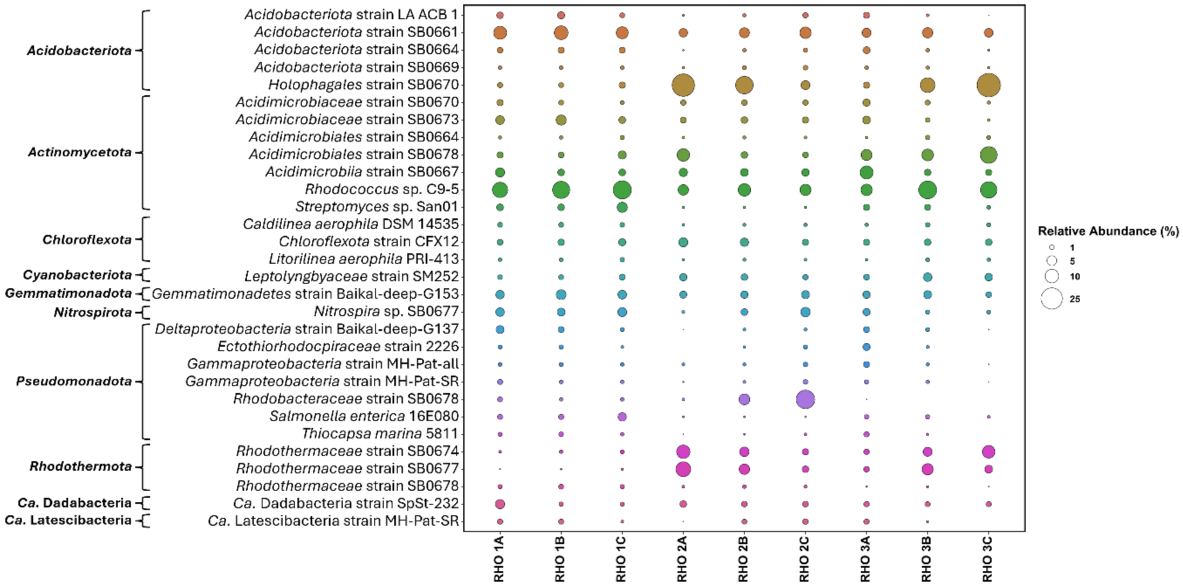

Figure S8: Bubble plot depicting the relative abundances of the 30 most abundant prokaryotic strains in three individuals and segments of *Rhopaloiedes odorabile*.

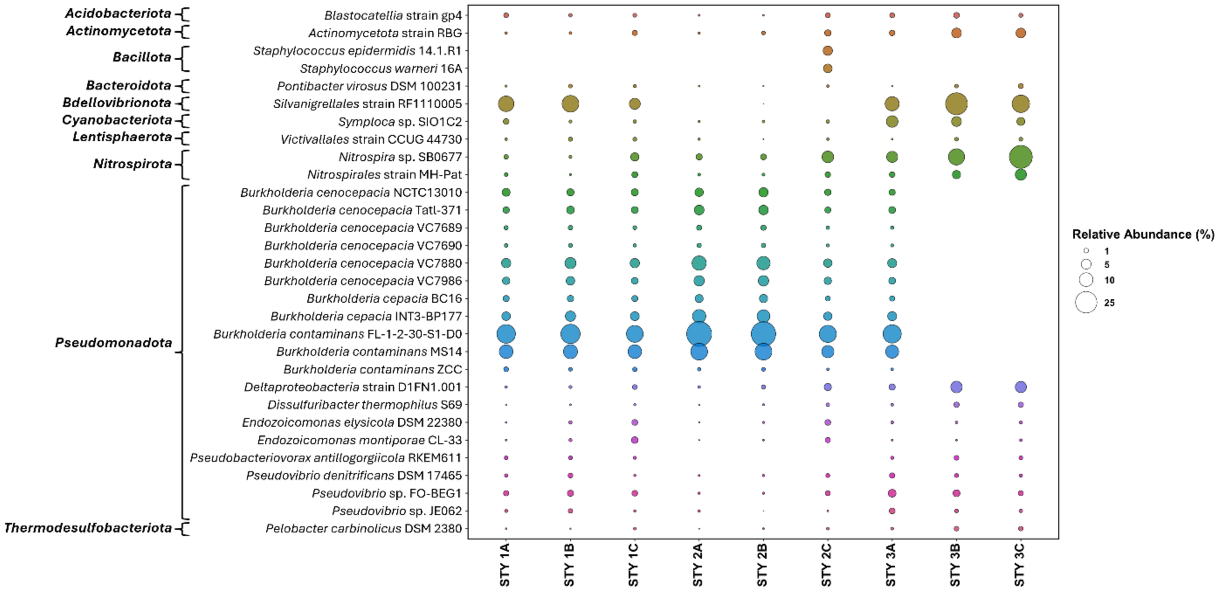

Figure S9: Bubble plot depicting the relative abundances of the 30 most abundant prokaryotic strains in three individuals and segments of *Stylissa flabelliformis*.
